# An Ideal

**Published:** 1881-07

**Authors:** W. P. Dickinson


### AN IDEAL.

*Read before the Iowa State Dental Society, May 11th, 1881.*

BY DR. W. P. DICKINSON.

"The shadow we pursue, still flees us,
Fast pacing as we faster pace."

In every matter of life, it is impossible for man to avoid having an ideal—it comes in the nature of the case. The very word indicates, that which pertains, or belongs to an idea; "existing in idea or thought" as of interior excellence, and which is, or may be hoped for or attained. It is also defined as something "visionary" or "existing in fancy or imagination only."

But it is not my purpose to inflict upon you an attempt, to discuss the abstract subject of ideality, nor will I presume that the ideal I shall try to present, will be fully attained in *our* day—perhaps you will not agree with me in believing it all desirable to *ever* be reached.

However, the thoughts here expressed, have come from time to time, when hoping for the "good time coming," when understanding and appreciation will take the place of error and misjudgment, and when success in life will not altogether be considered, to consist of the number of dollars one has accumulated, for some one else to squander after he has passed beyond the pleasure of counting them. I do not mean, in speaking of appreciation, that there is by any means a *total* lack of appreciation of our vocation, for there *are many*, who do fully and truly esteem our calling, but I am hoping that the time will be, when the *general* appreciation of the dentists services will be commensurate with their importance. It is a marvel that the dentist's mission is often so lightly thought of; a profession that "came into existence at the call of suffering humanity," and one "which is the necessary result of an enlightened and refined civilization," one too, which is "acting in concert with an advanced state of medical science."

It is true, many in the profession are at fault; that they sometimes seem to fail of a right apprehension of their duties and responsibilities; and through that failure, the public are not slow to conclude, that the whole may be rightly judged by them; whether this is a justifiable conclusion or not. I will not aver or contradict but *will* say, that the time of a correct estimate of us as a profession, will not come, before *we ourselves* have a high appreciation of our position, and prove by the manner in which we *pursue* our calling, that we are worthy of respect and confidence.

Specialties are among the necessities of this day of investigation and progress. The division of medical science is not however peculiar to our time; the simple fact of the impossibility of any single mind being able to grasp all that pertains to the nature and treatment of the many "ills that flesh is heir to," is a sufficient reason for such division, and the fact, that those who have attained celebrity in the medical profession, were specialists, confirms the wisdom of the plan. If it is impossible for one to know *all* that pertains to general medicine, it is also true that no one has yet appeared who knew *all* in any of the specialties, although in ours, there are some who seem to think they have reached the *ne plus ultra* of knowledge, if the course they maintain is any indication of their own judgment of themselves. And it is to be regretted, that so many who use the talismanic D. D. S. after their names, withhold their influence in reference to the work of establishing the profession in its proper place in the world. That which *stands* for education, *should* include refinment, and should also "beget a taste for like accomplishments in others."

Do not misunderstand me on this point and think I underrate the value of the *earned* degree. I am decidedly of the opinion, that no one should at this day, under any circumstance, enter upon practice without it, and will hail the day, when such a public and professional sentiment prevails, that will make it impossible for *any* person, to appear before the public as a dentist, before he is so qualified.

I would go further, and favor obliging even those now in practice, to choose between such a qualification, or discontinue business.

You may, by this time ask, what my dream for the future is, or what would in any measure contribute to the realization of *my ideal.* It has been said that "the trade of general improver is one that is apt to make us very odious to our fellows. Even if we can show demonstratively, that something better could be put in the place of something that is, we had better not say it." I shall therefore only speak for myself, If I succeed in awakening any r sponsive chord I will be gratified, if not, I will be sorry indeed.

It is but natural that to us, an ideal specialty or practice would be first to suggest itself. Have we yet reached the Pisgah, where we can look over and see, that at the outset, no practitioner will entertain the application of any one for studentship, whom he knows can never occupy a creditable position in the profession, by reason of his unfitness either by nature or education, or whose principal recommendation is, that "he is ingenious and handy with tools?"

Will the time come, when no one will presume to enter our ranks, "without the training and study essential to prepare him for his duties?" When it will be realized or fully comprehended to be a *crime* against society, to undertake a work for which he is unqualified, and when one does so, he will be rightly regarded as either ignorant, or devoid of principle or conscience; with respect to the ignorant aspirant for professional honor. I will just remark, that the more ignorant he is, the easier the pursuit of a profession appears.

It is amazing that when apprentices to the trades are expected to give from three to five years time to their acquirement, so many "shops" abound, where the same number of months, and in some instances even, weeks are deemed amply sufficient to turn out "*first class dentists*"; and a most astonishing fact concerning these establishments is, that they are countenanced and largely supported by those who certainly *ought* to know better. It is among this class, we find those who have so prominently displayed in their advertisements in the local papers and upon cards and handbills such legends as: "All (!) operations carefully performed and warranted," "Satisfaction guaranteed," etc. A

prominent attorney once remarked to me, that he had no idea a library was at all necessary, or that one to become a dentist, was obliged to devote any time to study, but supposed that to master the dental art the serving of a sort of apprenticeship for a few months accomplished the whole matter. How truly it has been said, that " He who speaks of a *doctor*, does not always speak of a *learned* man, but only of one who *ought* to be learned."

When the time comes that dentists will be less mercenary and devote more time to study and investigation, and less to the means of making the most money in the least possible time, without care as to the *real* interests of their patients; when no one will fear there is danger of knowing too much, and when the fact will be recognized, that to avert disease, he must be able to discern the indications of its appearance, and that to treat disease, he must not be ignorant of its nature, or incapable to investigating its cause.

I am decidedly of the opinion, that success in its true sense, will not be the portion of any one, who is not an enthusiast in his specialty, and it may be pertinent to add that the real students and investigators in our ranks are far too few.

When the time comes that *character*, which is the embodiment of all the virtues of honesty, faithfulness, purity of mind and life, and all that is good, shall be deemed as *requisite*, by the public in him who shall occupy the place of dental adviser to their families. How often is the dentist placed upon his honor, in the treatment of his patient, and if not an honorable men, who can measure the consequences?

When will the time be, that as we all expect the courtesy of cleanliness in those who apply for our services, we will cease our persistence in trampling under foot, the requirment of the same courtesy from us? I allude especially to the use of tobacco and intoxicating liquors. Prof. Watt, in speaking of this subject says:—" Many strongly scented substances, when taken into the stomach, pass into the circulation and are exhaled with the perspiration * * * * The odorous principles of garlic, onions and tobbacco, etc., are familiar illustrations of this fact. * * * * And the chewing and smoking of tobacco are often the means of introducing the most offensive smells into the dental office. When introduced by the patient, he should be charged double price for each operation performed, when by the operator, the patient should leave the office without hesitation or ceremony.

When the time comes that "society" will not be *used* as a means of professional advertisement and advancement, when merit and capability will be sufficient to insure a livelihood, and when the profession shall consider all such means as a sacrifice of professional dignity and standing; we must not forget, that "while we are members of the profession, we are also members of society at large; and as membership always implies corresponding duties, we are required, not as a profession, but as individuals to bear our part in the care and toils of life; and as we are proud to call ours a liberal profession, it becomes us to devise liberal things; society always expects more from the enlightened and educated than from those whose opportunities and attainments are more limited; and this is right."

When the time comes, that the local and state dental societies will be more generally attended and supported, when no one can be found to assert that they see little use or benefit in them, and especially the papers read, particularly any elucidation of such unimportant (?) subjects as Histology, Physiology, Pathology or Hygiene, who think this all useless, or a waste of time, and whose highest conception of the duties of the profession is, that our minds should not venture beyond the details of filling teeth, or the work of the laboratory, and who add that such things were not considered necessary when they "learned their trade."

When the day arrives, that the words "quackery" and "malpractice" will have gone out of use, except when referring to the past. Society demands that the physician whose incompetency or negligence causes the loss of an eye or deformed and crippled limb, should be punished, and justly too. Why should the dentist, who wilfully and wickedly sacrifices a set of natural teeth, or a single tooth even, not be amenable to the same law? The startling consequences of the quack's practices are so common, I will simply mention the subject without discussion. The question has been asked "will quackery ever die of starvation?" I answer, not until the people *can* and *will* discriminate in favor of the qualified practitioner as against the incompetent; and while they will persist in risking their health and lives even, for the sake of false ideas of economy, the hope of the speedy demise of the monstrous system is not flattering.

I have thus outlined a few things in the profession, which would help answer the requirement of *my ideal;* there are still others, which pertain to the public.

When the time comes that the dental adviser will be selected, having in view his general good reputation, habits, associations and character, as previously indicated, as well as for the known results of his skill, and not through personal motives alone, or because he may have "danced with me when I was a girl"; when no one will assert their opinion, that the greater part of the dental services rendered, are unnecessary, because *they* cannot *see* the necessity for them, and for that reason are suspicious of being taken advantage of; or because they may at some remote time have had unskillful service performed, which failed of its purpose, consider "dentistry a failure."

When we will not be called upon and be expected to do impossibilities in the way of restoring health to organs beyond the hope of salvation, through the neglect of their possessors, who come with the sacrilegious remark that they "do not see why our teeth were not made to last a life-time." When people will not expect a degree of perfection or durability of dental operations, above everything else made by the hand of man, and when they will learn that the *best* and finest work *will not* preserve teeth, in defiance of their own abuse and carelessness.

When the teeth of children will not be allowed to "go to destruction" because they have the choice whether attention shall be given or not; or because of morbid sympathy for the child's present feeling. And we are directed to "do nothing if it is going to hurt;" or it is remarked: "I would rather have her lose her teeth than be late at school." When such embarrassments as these are all removed, it will be much easier to promote the preservation of the temporary teeth than at present; and the permanent teeth will also be more fully appreciated.

When the patient will never defraud the dentist, by neglect to keep engagements, and when no one will for a moment presume to entertain the thought, that their dentist *must* certainly be under a debt of gratitude, *for the privilege of serving them in his professional capacity.*

When in respect to fees, a better understanding will obtain; when skill will meet its just reward, and when quacks may be indicted and punished, both for the result of their malpractices and for obtaining money under false pretenses.

"People, generally, are not aware of the importance of a fee. A physician can make a more correct diagnosis, and his remedies will operate much more beneficially, where he is certain of being paid, than where he proceeds without reference to the recompense of reward. A fee inspires the practitioner with confidence in himself, and faith in his remedies, which is almost as important as the exercise of faith on the part of the patient. An eminent English physician once remarked "that he never felt his own pulse nor looked at his tongue in the glass, without unconsciously slipping a guinea from one pocket into the other." When this matter is better understood, and it devolves upon the profession to make it so. "Those who wish to derive the full benefit of advice will bear in mind the necessity of the fees." Then, too, will be the time when we will cease having calls, "just to see if any thing needs to be done," and after devoting perhaps an hour of valuable time to the examination of the case, receive the comforting assurance of a visit at some future time. This pernicious custom should be broken up, and we ourselves are at fault, that it has *not* been, ere this, for there is neither sense nor justice in it. It is a *real* service done, and one which is seldom paid for, either directly or indirectly. The more careful and conscientious the examination and estimate, the better opportunity is afforded some unscrupulous rival, to console the patient with the assertion that "but little *really* needs to be done now," and consequently does that "little," the victim not thinking that 'the dentist who will rob an honest fellow-practitioner by under-bidding, will rob the patient by doing inferior work.'"

The ideal practice cannot be, before it is understood that *good*

services *can not* be rendered for inadequate remuneration, when it will be remembered that the *best*, one can obtain is the cheapest, and when no one will themselves place a value upon services on the basis of so much material used, or that services for children should be charged for proportionately less than the same done for an adult, when the fact is, the fee should be larger. An amusing instance of the estimation in which a patient held a dentist's efforts is told by an Eastern practitioner, who was called up one night, to treat a case of hemorrhage, following the extraction of a tooth by another dentist; having subdued it, a drachm bottle of nut galls was given, to use in case of recurrence of the trouble. Months after, when a bill of two dollars was sent in, word was returned that it was too much *to pay for so small a bottle of medicine.* This same writer also says: "Dentists are not allowed a *professional* fee, it is almost without exception considered to be an extortion, unless it bears some reasonable relation to the cost of the material used."

Happily, there is a bright side to dental practice. There *are* those who see that the relieving of the suffering and the restoring health to the diseased is an honorable and useful vocation, who do not hesitate to encourage us by a just compliment upon our services, who promptly pay their indebtedness, and who afterwards co-operate with us in our efforts to avert further pain and trouble. Among the choicest fruits of a faithful pursuit of the profession, as expressed by another, "are self-approval, appreciative patronage and pecuniary emolument."

I have thus tried to give you the outlines of *an ideal* future practice; I have tried as Ruskin advises to say what I had to say "in the plainest possible words," and also to deal in downright facts, which we need at the present day more than anything else." I have not forgotten the proverb, "Follow not truth too near the heels lest she dash out your teeth."

There is much yet to be said, but I will not undertake to pursue the matter further. In closing, let me indulge the hope that the day is almost at hand, when we will be "measured by what we can do, rather than by what we can tell." When competition will be encouraged, but that it must be in quality of work rather than smallness of fees; when all will be ready to see the good in others and in their works, and will be glad to so testify when an opinion is requested, for by thus doing, the public at large will be inspired with confidence in that which we have confidence in ourselves.

